# Supplementary figures and images for: Disparate temperature-dependent virus–host dynamics for SARS-CoV-2 and SARS-CoV in the human respiratory epithelium
Source: PLoS Biol. 2021 Mar 29;19(3):e3001158. doi: 10.1371/journal.pbio.3001158 (PMC8032198; doi:10.1371/journal.pbio.3001158)

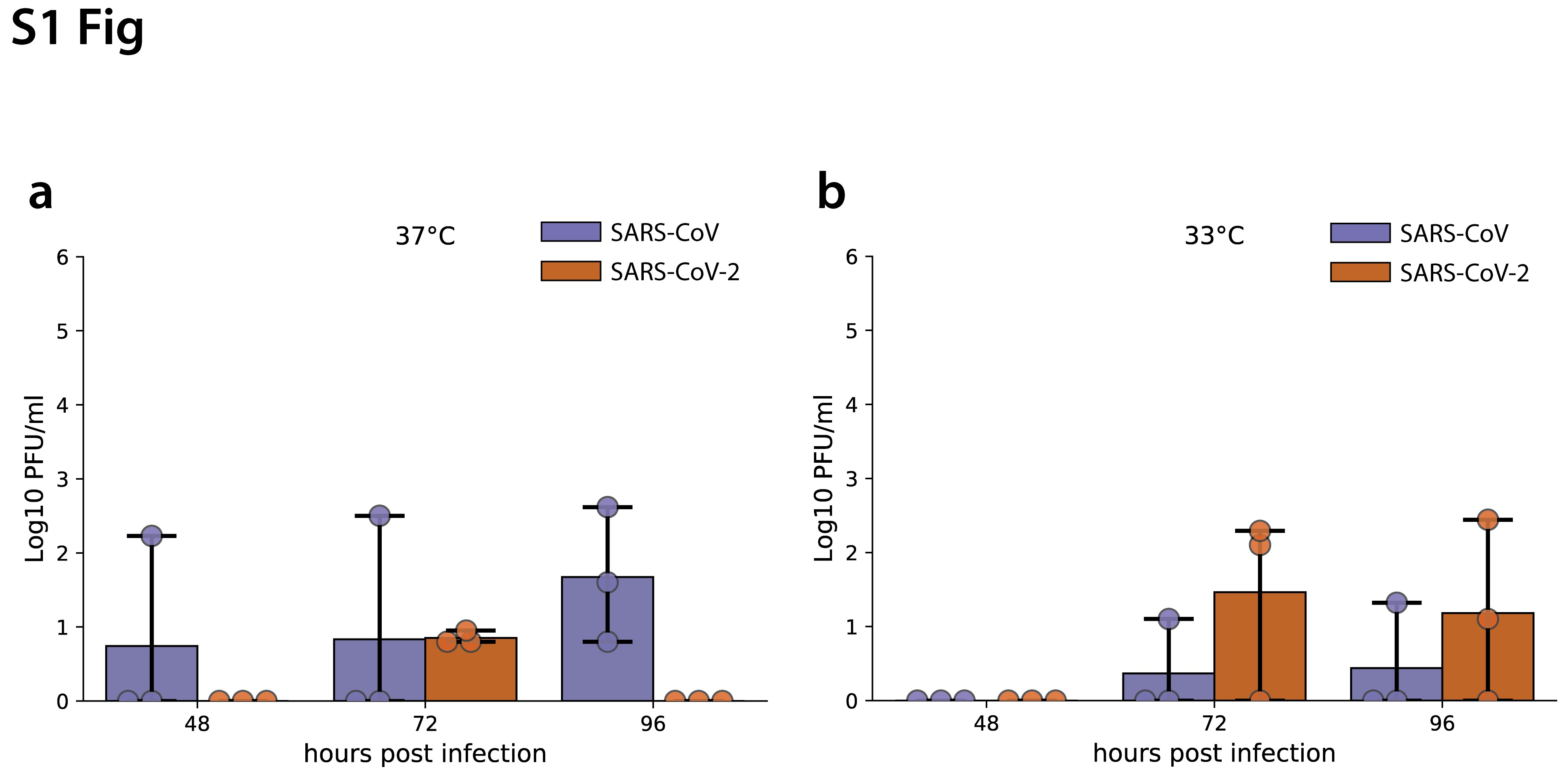

Supplement: S1 Fig — Well-differentiated hAEC cultures were infected with SARS-CoV and SARS-CoV-2 using 30,000 PFU or remain uninfected (mock) and were incubated at 37°C (a) or 33°C (b). Inoculated virus was removed at 1 hpi, and the apical side was washed. Cultures were further incubated at the indicated temperature. At the indicated time post infection, virus release in the basolateral compartment was assessed by plaque titration (a, b). Data represent the mean 2 two replicates. The data underlying this figure are found in S1 Data. hAEC, human airway epithelial cell; hpi, hours post infection; PFU, plaque-forming unit; SARS-CoV, Severe Acute Respiratory Syndrome Coronavirus; SARS-CoV-2, Severe Acute Respiratory Syndrome Coronavirus 2. (TIF) [file pbio.3001158.s001.tif]

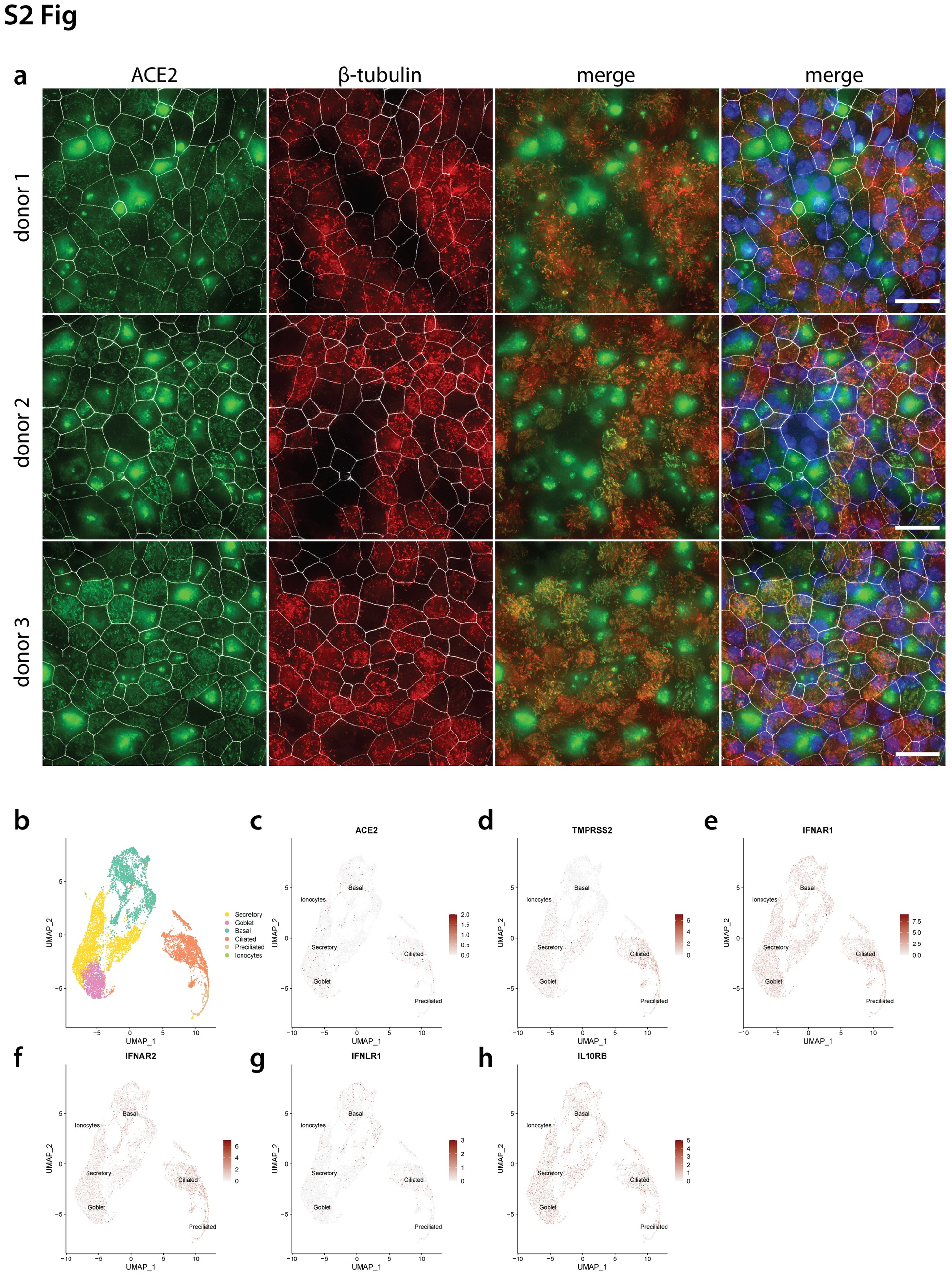

Supplement: S2 Fig — Immunofluorescence analysis of ACE2 receptor distribution in unexposed well-differentiated hAEC cultures (a). Unexposed well-differentiated hAEC cultures were fixed and processed for microscopy analysis using antibodies against ACEC2 (green), β-tubulin (cilia, red), ZO-1 (tight junctions, white), and DAPI (blue). Representative z-projections of 3 donors are shown. Scale bar, 20 μm. Unexposed well-differentiated hAEC cultures from different human donors were used to perform scRNA-seq analysis. The UMPA plots shows the dimensional reduction of the 8,128 cells belonging to either basal, goblet, secretory, preciliated, and ciliated cell population (b), as well as the relative expression level and distribution of ACE2 (c) and TMPRSS2 (d), respectively. In addition, the respective relative expression level and distribution of both the IFN-alpha/beta receptor alpha and beta chain, IFNAR1 (e), IFNAR2 (f), and the type III IFN receptor complex IFNLR1 (g), and IL-10RB (h). ACE2, angiotensin-converting enzyme 2; hAEC, human airway epithelial cell; IFN, interferon; scRNA-seq, single-cell RNA-sequencing; UMAP, Uniform Manifold Approximation and Projection. (TIF) [file pbio.3001158.s002.tif]

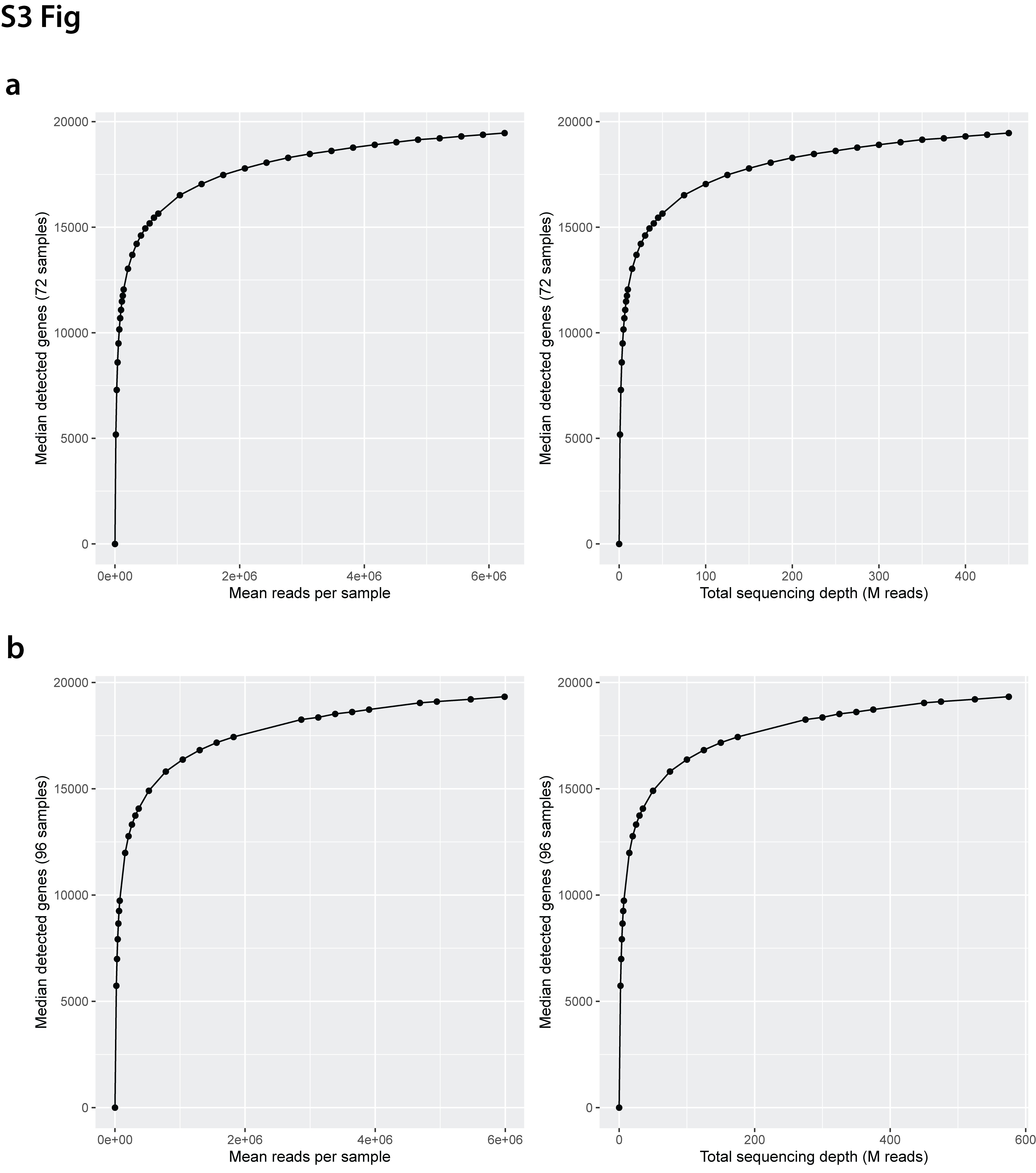

Supplement: S3 Fig — Sequence saturation graphs from 2 individual BRB-seq libraries, encompassing 72 (a) or 96 (b) samples from 3 or 4 independent biological donors, respectively, illustrating the average number of reads (x-axis) and median number of detected genes per library (y-axis; >1 read) across samples (left) and total sequencing depth (right; M = Million). BRB-seq, Bulk RNA Barcoding and sequencing. (TIF) [file pbio.3001158.s003.tif]

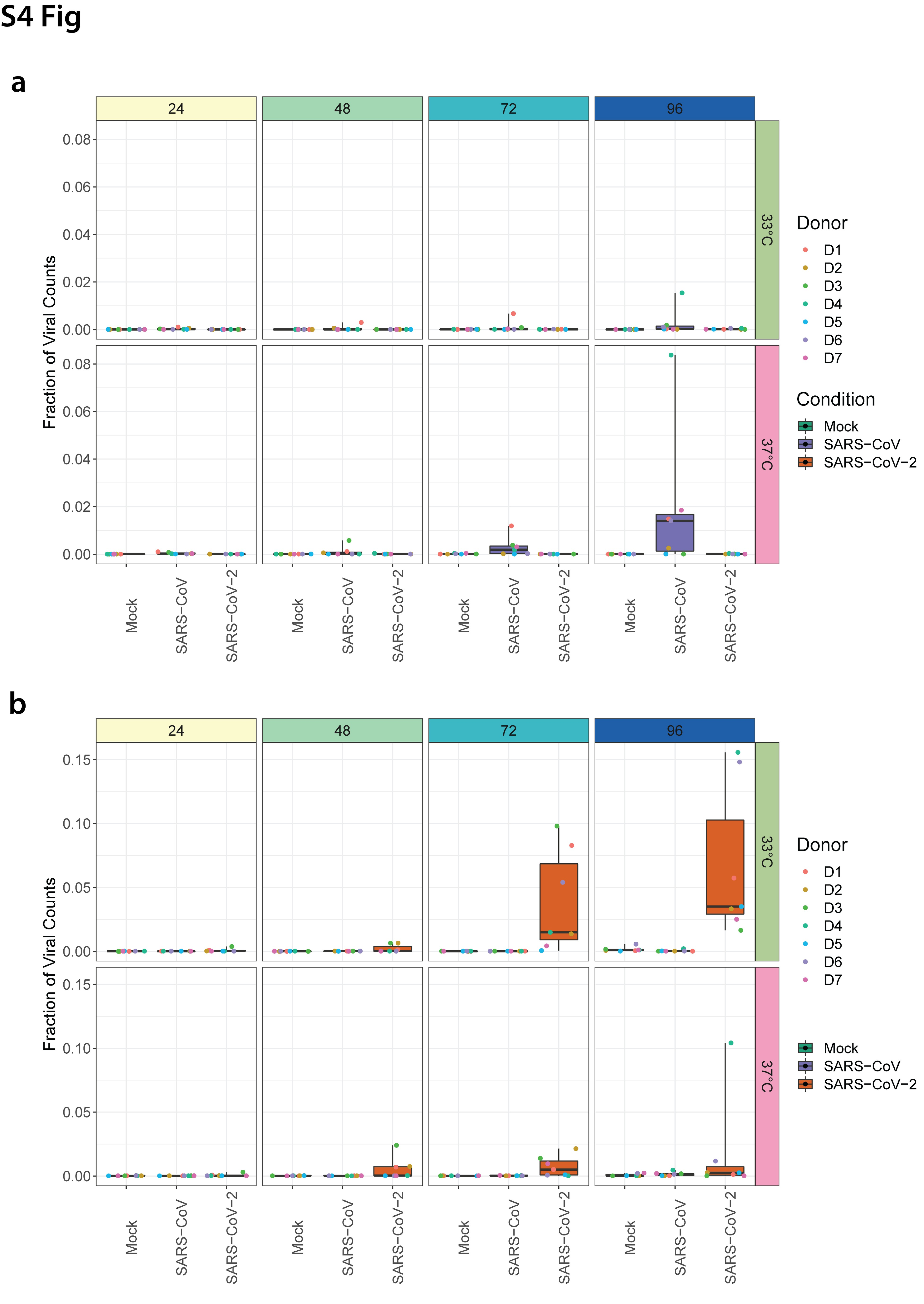

Supplement: S4 Fig — Boxplots illustrating the distribution of the fraction of SARS-CoV (a) and SARS-CoV-2 (b) specific viral reads among the total fraction of sequence reads at the different time points and temperature conditions in the respective individual untreated (Mock), SARS-CoV, and SARS-CoV-2 samples (colored by donor). hAEC, human airway epithelial cell; SARS-CoV, Severe Acute Respiratory Syndrome Coronavirus; SARS-CoV-2, Severe Acute Respiratory Syndrome Coronavirus 2. (TIF) [file pbio.3001158.s004.tif]

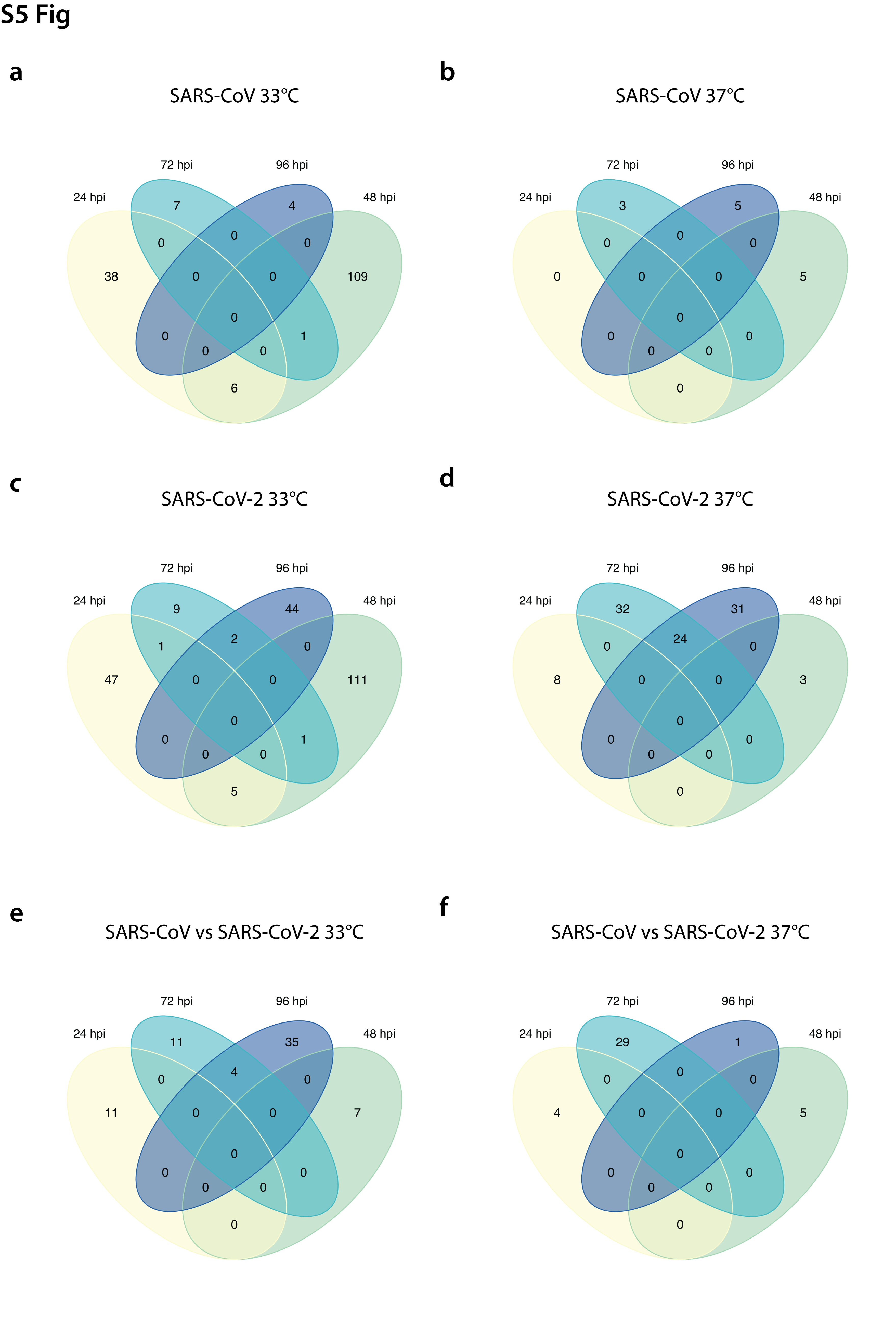

Supplement: S5 Fig — Venn diagrams showing the overlap of DEG in SARS-CoV or SARS-CoV-2 infected hAEC cultures among the different time point and temperature conditions (a-d), and SARS-CoV-2 contrasted to SARS-CoV at the different time point and temperature conditions (e, f). DEG, differentially expressed genes; hAEC, human airway epithelial cell; SARS-CoV, Severe Acute Respiratory Syndrome Coronavirus; SARS-CoV-2, Severe Acute Respiratory Syndrome Coronavirus 2. (TIF) [file pbio.3001158.s005.tif]

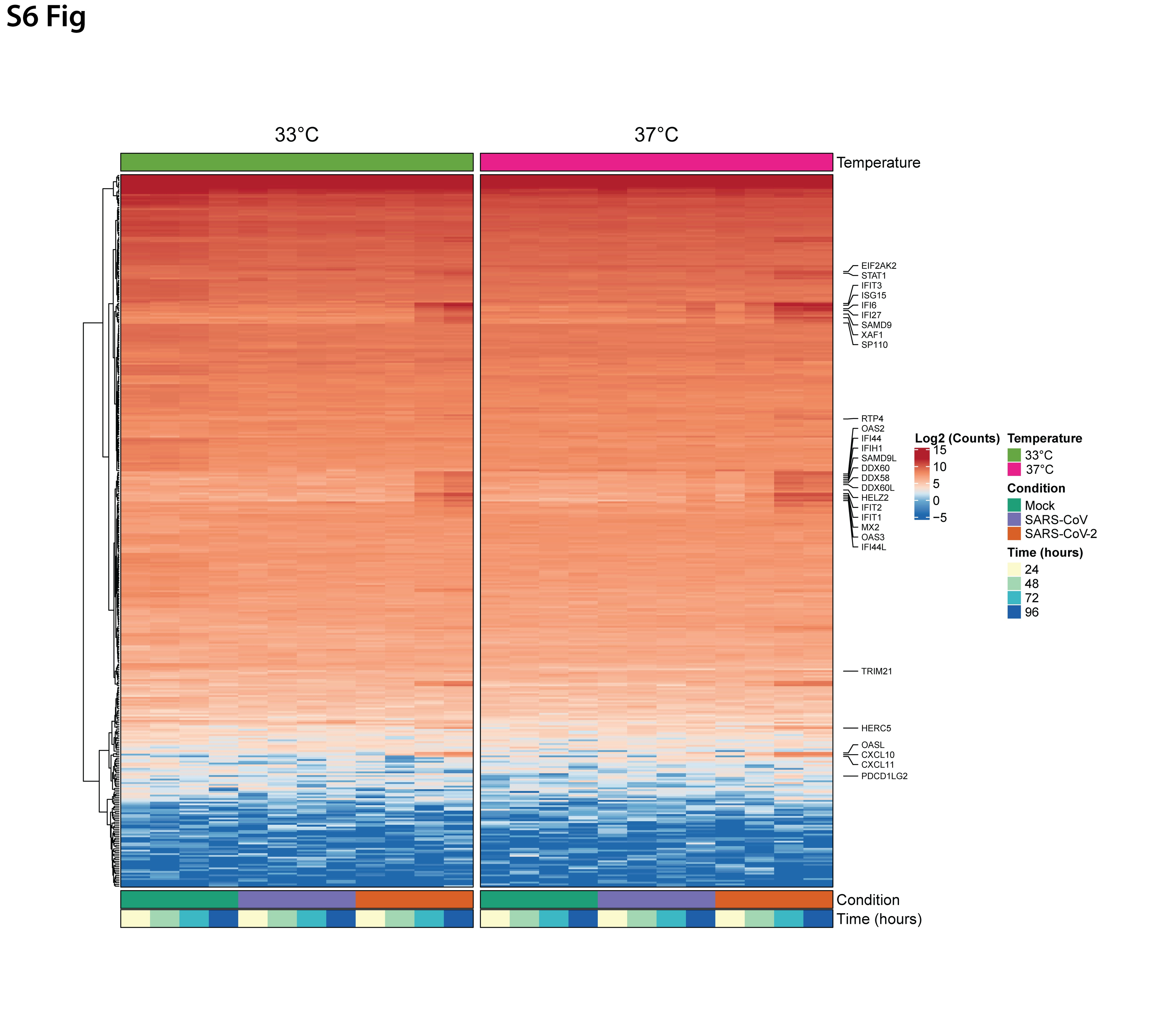

Supplement: S6 Fig — A heatmap illustrating the hierarchical clustering of the expression levels of 401 unique DE genes identified in the pairwise comparison of SARS-CoV and SARS-CoV-2 infected hAEC cultures compared to uninfected hAEC cultures (Mock) at different time points and incubation temperatures. Expression levels for individual temporal DE genes are shown in rows as the log2 mean normalized counts for 7 human donors stratified by condition, temperature, and hours post infection (columns; representative colors shown in legends). The 29 temporal DE genes unique for cluster 1 are shown on the right (y-axis). DE, differentially expressed; hAEC, human airway epithelial cell; SARS-CoV, Severe Acute Respiratory Syndrome Coronavirus; SARS-CoV-2, Severe Acute Respiratory Syndrome Coronavirus 2. (TIF) [file pbio.3001158.s006.tif]

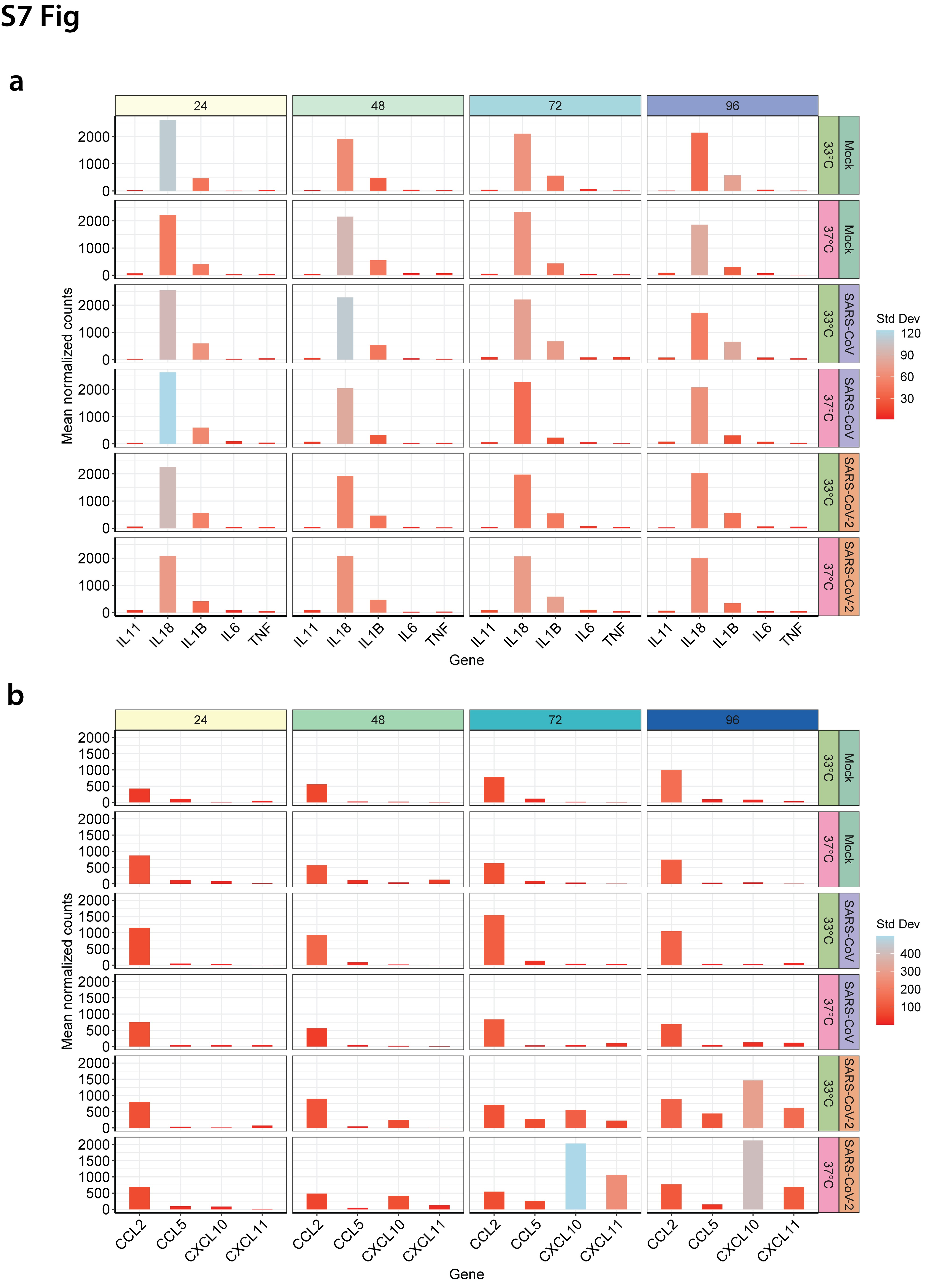

Supplement: S7 Fig — Bar graph illustrating the mean normalized expression levels over time for the cytokines IL11, IL18, IL1b, and TNF (a), or the chemokines CCL2, CCL5, CXCL10, and CXCL11 (b), at the respective temperatures for Mock (uninfected) hAEC cultures (top two panels) and for SARS-CoV (middle two panels) and SARS-CoV-2 (bottom two panels) infected hAEC cultures. Bars were adjusted in color to illustrate the respective SD among donors. hAEC, human airway epithelial cell; SARS-CoV, Severe Acute Respiratory Syndrome Coronavirus; SARS-CoV-2, Severe Acute Respiratory Syndrome Coronavirus 2; SD, standard deviation. (TIF) [file pbio.3001158.s007.tif]
