# Supplementary material for: Disparate temperature-dependent virus–host dynamics for SARS-CoV-2 and SARS-CoV in the human respiratory epithelium
Source: PLoS Biol. 2021 Mar 29;19(3):e3001158. doi: 10.1371/journal.pbio.3001158 (PMC8032198; doi:10.1371/journal.pbio.3001158)
Supplement: Attachment — Submitted filename: Rebuttal Letter Jan 2021.pdf [file pbio.3001158.s014.pdf]

**Reviewer 2**

1. Figure 3 clarifications
   (i) Fig 3a-Can the authors clarify what the main functional categories of each gene cluster in the Venn diagram are. For example, aside from ISGs, are there other functional categories of enriched genes in the overlapping and non-overlapping gene sets?

We thank the reviewer for this excellent suggestion. A supplementary table (S2 Table) now provides the details of the genes included in Fig 3a and their functional classifications are now illustrated in Fig 3b.

The revised text now includes the following sentence at lines 196 - 201: *"Pathway enrichment analysis of individual gene clusters revealed that the 43 DE genes shared between SARS-CoV and SARS-CoV-2 infections at 33°C were predominantly associated with eukaryotic mRNA translation pathways, whereas the specific genes for SARS-CoV infections at 33°C were mostly related to chemokine signalling pathways (Fig 3b, S2 Table). In contrast, DE genes identified for SARS-CoV-2 infections at both 33°C and 37°C were mainly associated with the host antiviral response (Fig 3b)."*

(ii) Fig 3d- It is not clear how the information in this figure was derived and what the figure is showing.

Figure 3d, that illustrated the overlap of the number of genes with their respective enriched pathways, was deleted from the revised manuscript since it was redundant with the inclusion of the improved transcriptome analysis now shown in Fig 3, and Suppl Fig 3 - 7.

2. RNASeq data vs. growth curve: Can the authors clarify how many donors/replicates per condition were used for RNASeq. In figure S5, more viral reads are shown at 37C than 33C for SARS-CoV – this doesn't seem representative of the data in Figure 1. Given the variability among donors shown in Fig 1, it would be helpful if the authors can indicate which donor(s) were used for RNASeq, so that the reader can better interpret how generalizable the results are.

We apologize for this lack of clarity. In the revised manuscript, SARS-CoV and SARS-CoV-2 replication at 33°C and 37°C from 3 donors were complemented using cells derived from 4 additional human donors. In total, cells from 7 donors were used for the assessment of viral growth kinetics. The legend of Figs 1a and 1b at lines 516 - 518 clearly states that *"data represent the mean ± 95% CI of hAEC cultures from seven different human donors. Individual points represent the average of two technical replicates".*

In addition, to provide data at a greater sequencing depth, the three initial samples were re-sequenced together with the samples derived from the 4 additional donors. In total, 7 donors were included in the transcriptome analysis, which are identical to those used for the viral kinetic experiments

In FigS4 (formerly Fig S5), the mean value for SARS-CoV 37°C was influenced by one single donor displaying elevated reads. The figure has been updated with boxplots illustrating the distribution of the data, and individual datapoints, from the seven donors to better visualize the variation between samples.

**3.** SARS-CoV: The manuscript's stated goal is to compare the host response to SARS-CoV and SARS-CoV-2, but the authors do not describe which genes or pathways were enriched in the transcriptome analysis of SARS-CoV infected cells in the results or discussion.

We thank the reviewer for this relevant remark. As stated in the text (e.g. at lines 232-234, 265-267, 294-295), Figs 3a-3e robustly show that, in agreement with the available literature, SARS-CoV does not induce substantial transcriptional changes in infected cells nor upregulation of interferon signalling pathways (Figs 3b and 3e).

4. Also, as mentioned previously by reviewer 1, can the authors comment on the relatively low amount of replication observed for SARS-CoV in this study (at both temperatures)? For example, based on the literature are sites other than the conducting airways thought to be the major target cells of SARS-CoV (e.g. alveolar epithelia?)

As already detailed in the previous answer to reviewer 1, SARS-CoV titers increased 10 to 100-fold during the course of infection in human airway epithelial cell cultures derived from primary human tracheobronchial tissues. In line with these results, independent studies using hAECs and an identical SARS-CoV stain obtained from a reverse genetic clone (DOI: 10.1128/mBio.00611-12 and DOI: 10.1038/s41598-018-33487-8) resulted in very similar outcomes.

5. Since the initial submission, several studies have been published regarding the host response to SARS-CoV2. It would be relevant for the authors to briefly discuss how the findings presented here relate to other published results and provide new insights.

We thank the reviewer for the comment, and we agree. Indeed, the main text (lines 319-331) now relates our findings concerning host responses to several published results over multiple experimental models. This comparison revealed that our results are in line with the findings of other groups on a functional level e.g., the consistent mounting of an interferon response and the upregulation of chemokines, while being represented by slightly different individual genes. In addition, we highlight the new insights provided by our comparison of viral replication and antiviral host responses between 33°C and 37°C.

**Reviewer #3 (Remarks to the Author):**

I have read all three reviews and the corresponding answers from the authors. I have also read the revised manuscript. I remain unconvinced of their presentation, and I find the

current manuscript plagued with inconsistencies. The claim of differential sensitivity to interferon is now dropped from the original manuscript (although still mentioned in the abstract!). The use of the transcriptome analysis is non-supportive of any of the claims. As it stands, all that can be said is that in this in vitro model, using just 1 strain from each virus, there is 1 log more SARS-CoV2 replication. Whether this is sufficient to support the in vivo claims is, in my view, questionable.

We regret to read the reviewer's perspective of our scientific work and are very surprised that the concerns regarding the transcriptome analysis were not raised by the reviewer earlier during the peer review process. We were particularly surprised by these comments as after the initial constructive feedback, we include a significantly deeper sequencing depth (4 Mio to 12 Mio reads), as well as four additional donors (n=3 to n=7) in the revised version. Furthermore, we previously clarified to the reviewer that the reconstructed reverse genetic clone of SARS-CoV Frankfurt-1 strain used in our experiments does not carry a truncated ORF7b. We address these aspects in further detail in a point-by-point response to the more constructive confidential comments to the editor, that were graciously provided to us by the editor from Nature Microbiology.

**Reviewer 3 (Remarks to the Editor)**

1. The abstract still claims that CoV2 "displayed higher sensitivity to type I and type III IFNs" even though the main text, the figures and the statistics (and answers to reviewers) appear to walk away from that notion.

We agree with the reviewer and apologize for this unfortunate omission. The abstract now clearly states that both SARS-CoV and SARS-CoV-2 are highly sensitive to type I and type III IFNs.

2. The manuscript (abstract, discussion) claims that "These data reflect clinical features of SARS-CoV-2 and SARS-CoV, as well as their associated transmission efficiencies, and provide crucial insight on pivotal virus - host interaction dynamics" – this is not what this manuscript reflects. There is no information on clinical features or on transmissibility here.

Our data do reflect characteristic features observed during the clinical course of SARS-CoV and SARS-CoV-2 infection. Indeed, high viral replication in the upper respiratory airways (nasopharynx) was reported in patients early after infection with SARS-CoV-2. Moreover, late and aberrant innate immune activation in later phases is suspected to contribute to the pathologies associated with SARS-CoV-2 infections. In strong contrast, SARS-CoV was demonstrated to preferentially replicate in an uncontrolled manner (i.e., initially evading the immune restrictions) in the lower respiratory tract, ultimately leading to immune cell infiltration in the lungs associated with tissue damage.

To avoid any confusion, we rephrased our statements throughout the manuscript (e.g. lines 43-45, 295).

3. The manuscript builds on the different kinetics of 1 strain of SARS-CoV-2 and 1 strain of SARS-CoV. How generalizable would this be to other strains, to other huCoV. Would the authors speculate that these experiments reflect the true biology of CoV2?

We thank the reviewer for this raising this interesting question. Both SARS-CoV and SARS-CoV-2 strains used in our study are representative prototypes of the viral strains having circulated or are currently circulating in the human population, respectively. The examination of SARS-CoV and SARS-CoV-2 variants and whether their replication is affected at different temperatures is certainly of high interest yet lies beyond the scope of this work.

4. The discussion on SARS-CoV strain Frankfurt-1 (GenBank FJ429166) remains confusing. The Discussion indicates "Another factor that may have influenced our results is the 29-nucleotide truncation in the ORF8 gene of SARS-CoV Frankfurt-1, which was maintained in the SARS-CoV lineage that initiated the international spread of SARS-CoV" . However, in the answer to the reviewers, the authors indicate that the SARS-CoV strain Frankfurt-1 used in the study had the deletion in ORF7b corrected. Could this be re-clarified?

The reconstructed reverse genetic clone of SARS-CoV strain Frankfurt-1 (GenBank FJ429166), which was also used in this study, is a prototype SARS-CoV strain that circulated in the human population in 2002/03 (doi: 10.1186/1743-422X-6-131). In this clone, the artefact ORF7b truncation acquired upon isolation and passaging in cell culture was corrected. Independently from this, a deleterious ORF8 truncation in SARS-CoV was acquired along the initial human-to-human transmission chain during the SARS-CoV 2002/2003 outbreak and was subsequently associated with a loss of fitness (https://doi.org/10.1038/s41598-018-33487-8). In our discussion, we raise the question whether an intact ORF8 in SARS-CoV, or inversely a truncated ORF8 in SARS-CoV-2, would impact their replication efficiencies at different temperatures. This remains to be formally addressed experimentally but lies outside of the scope of this work. The corresponding section in the discussion has been explicated and now reads (lines 304-314 ): *"Another factor that may influence the temperature-dependent replication phenotype is the different form of the ORF8 gene in SARS-CoV Frankfurt-1. The 29-nucleotide deleterious truncation in the ORF8, which is associated with a loss of fitness, was acquired during the initial human-to-human transmissions and was maintained in the SARS-CoV lineage that is at the origin of the international spread of SARS-CoV [35]. Therefore, besides comparing the replication of different SARS-CoV ORF8 variants at 33°C and 37°C, it would be equally compelling to assess the phenotypic influence of similar truncations in the ORF8 gene of SARS-CoV-2, especially since several SARS-CoV-2 isolates*

*bearing a 382-nucleotide deletion truncating the ORF8 gene have been detected [36]. Such SARS-CoV-2 ORF8 variants can be readily engineered using the reverse genetic systems that were recently established for SARS-CoV-2 [32,37,38]."*

5. The presentation of the transcriptome data is very difficult to follow – and as it stands, inconclusive. The data seem to describe differences across strains, times and temperature without a clear message. It does not seem to contribute to clarity in the message, and does not seem to provide mechanistic insights."

The presentation of the transcriptome data has been improved and additional analyses have been implemented. This is reflected in Figs 3 and 4 as well as in S3-S7 Figs. The two paragraphs (starting at lines 171 and 242) have been improved and include the results from the latest analysis.

Molecular mechanisms involved in these phenotypes must be investigated separately as they lie beyond the scope of this work. The revised manuscript now also includes the following sentence at lines 272-275: *"However, whether more potent innate immune activation restricts SARS-CoV-2 replication at 37°C, or whether a distinct virus-host interplay favours SARS-CoV-2 replication at 33°C awaits to be formally determined."*
As well as, at lines 349-351 *"These data will likely be extended by additional mechanistic and functional in vivo studies delineating the efficacy of antiviral host responses triggered by SARS-CoV and SARS-CoV-2 infections, as well as deciphering the influence of virus-encoded antagonists and physical parameters."*
